# Amygdalin synergizes with the TNF-α monoclonal antibody infliximab to modulate HSP90 and related necro-inflammatory/oxidative stress pathways in a rat model of hepatic I/R

**DOI:** 10.1007/s00210-025-04866-6

**Published:** 2025-12-17

**Authors:** Reem A. Mohamed, Mai El-Sayed Ghoneim, Rasha A. Tawfiq, Nermein F. El Sayed

**Affiliations:** 1 Department of Pharmacology, Faculty of Pharmacy, October University for Modern Sciences and Arts (MSA), 26 July Mehwar Road Intersection With Wahat Road, 6th October City, Egypt; 2 Department of Pharmacology and Toxicology, Faculty of Pharmacy, University of Sadat City (USC), Sadat City, 32897 Egypt; 3 Department of Pharmacology, Faculty of Pharmacy, The British University in Egypt, Cairo, Egypt; 4 Drug Research and Development Group (DRD-G), Research Center of Excellence, The British University in Egypt, Cairo, Egypt; 5 Department of Pharmacology and Toxicology, Faculty of Pharmacy, Ahram Canadian University, Giza, Egypt

**Keywords:** Amygdalin, Hepatic ischemia/reperfusion, Heat shock protein 90, Infliximab, Necroptosis, NF-κB

## Abstract

Hepatic ischemia/reperfusion (I/R) injury remains a major limitation in liver surgery and transplantation. Amygdalin, a natural glycoside, is known for its antioxidant, anti-inflammatory, and hepatoprotective properties, but its combination with conventional agents is not studied. Accordingly, this study explores a combinatorial approach using a small dose of amygdalin (5 mg/kg) and low-dose infliximab (1.5 mg/kg), a tumor necrosis factor (TNF)-α inhibitor, as a pre-treatment to alleviate I/R-induced liver injury. It also investigates the unexamined role of heat shock protein (HSP)-90 in the modulation of necro-inflammatory pathways during hepatic ischemia/reperfusion injury. Rats were divided into six groups: sham, I/R, infliximab (1.5 and 3 mg/kg), amygdalin (5 mg/kg), and a combination of infliximab (1.5 mg/kg) with amygdalin. Treatments were administered intraperitoneally for three days before I/R induction. Both monotherapies and the combination significantly reduced hepatic expression of HSP90, TNF-α, and phosphorylated-mixed lineage kinase domain-like protein (*p-MLKL*), while restoring oxidative balance as evidenced by modulation of nuclear factor erythroid 2-related factor 2 (Nrf2), malondialdehyde (MDA), and superoxide dismutase (SOD). The combination therapy additionally suppressed nuclear factor kappa B (NF-κB) in a synergistic manner and enhanced Nrf2 expression compared to amygdalin monotherapy. Correlation analysis revealed strong associations between markers of necroinflammation and oxidative stress, reinforcing the mechanistic interplay between these pathways. HSP90 emerged in the current study as an intermediate modulator linking necroinflammation to oxidative responses. Importantly, the combined therapy exerted a synergistic effect by effectively modulating HSP90 and associated signaling cascades, underscoring its potential as a superior therapeutic strategy.

**Supplementary Information:**

The online version contains supplementary material available at 10.1007/s00210-025-04866-6.

## Introduction

Hepatic ischemia/reperfusion (I/R) injury remains a significant clinical challenge, especially in liver transplantation and trauma. It occurs when the liver’s blood supply is temporarily interrupted and then restored, triggering a cascade of pathological events leading to inflammatory responses, increased oxidative stress, and cellular damage (George et al. 2024). Necroptosis, a programmed form of cell death, is implicated in the pathological process of hepatic I/R injury (Baidya et al. 2020; Li et al. 2021b). While having the same histological appearance as necrosis, necroptosis shares the activation signals of apoptosis. Three key protein molecules mediate it: receptor-interacting protein kinase 1 (RIP1), receptor-interacting protein kinase 3 (RIP3), and mixed lineage kinase domain-like protein (MLKL) (Wu et al. 2012). The interaction of these proteins results in pore formation in the cell membrane with the release of damage-associated molecular patterns (DAMPs), leading to an exacerbated secondary cascade of inflammation known as necroinflammation (Shi et al. 2019). The inflammatory mediators released from necroptotic cells include tumor necrosis factor-α (TNF-α), which re-triggers necroptosis in a vicious cycle of inflammation and ultimately hepatocyte death (Pinci et al. 2022). The role played by molecular chaperones such as heat shock protein 90 (HSP90) in the modulation of these necro-inflammatory pathways in hepatic I/R has not been previously investigated.

HSP90 is a highly dynamic molecular chaperone that can participate in the proteostasis of proteins (Chiosis et al. 2023). It interacts with other co-chaperones and protein clients to regulate the formation of multiprotein complexes through ubiquitination-mediated folding or unfolding decisions (Schopf et al. 2017; Biebl and Buchner 2019). Reports on the inflammatory effects of HSP90 in models of arthritis (Conte et al. 2015), colitis (Collins et al. 2013), and lung inflammation (Lilja et al. 2015) made it an interesting target for investigation in a hepatic I/R model.

Amygdalin, a natural compound derived from the seeds of bitter apricots (Figurová et al. 2021), was reported to reduce the severity of liver injury in experimental models of liver fibrosis (Wang et al. 2021), autoimmune liver disorders (Elsaed 2019), and acetaminophen-induced acute liver failure (Zhang et al. 2022); however, the protective effect of amygdalin against hepatic I/R injury and its molecular targets in this respect have not been previously investigated. Conversely, infliximab, a TNF-α inhibitor, is a well-established anti-inflammatory agent in both clinical practice (Smolen and Emery 2011), as well as experimental models of I/R injury (Yucel et al. 2015; Jawad et al. 2022).

Given the unexplored protective potential of amygdalin in liver injury induced by I/R, the present study aimed to investigate, for the first time, the possible protective effect of a small dose of amygdalin in an experimental model of hepatic I/R injury in rats and its hypothesized anti-necroptotic action. This was conducted by studying its effect individually and in combination with infliximab, focusing on the combined therapy as a therapeutic approach and HSP90 as a drug target in hepatic I/R.

## Materials and methods

### Animals

Eight weeks old Sprague Dawley female rats (150–24 g) were obtained from the animal unit of the British University in Egypt (BUE) (Cairo, Egypt). They were kept for 1 week to familiarize, bred in transparent standard plastic cages (3 rats/cage), at a temperature of 23 ± 2 °C and 60 ± 10% humidity with 12 h of dark/light cycles. Water and rat pellet diets were supplied ad libitum. Animals were randomly allocated to the different experimental groups, and sample analyses were performed by analysts blinded to the group assignments. The study protocol complied with the International Institutes of Health recommendations outlined in the Guide for the Care and Use of Laboratory Animals after the approval of the Research Ethics Committee guidelines of BUE (Approval No.: EX-2309) and adhered to ARRIVE guidelines.

### Drugs and chemicals

Infliximab was procured from Johnson & Johnson’s Janssen pharmaceutical company (New Brunswick, New Jersey, USA) and was dissolved in saline for injection. Amygdalin was purchased from Sigma-Aldrich (St. Louis, MO, USA,cat#: 29,883–15-6) and was prepared in saline for injection. All injectable solutions were prepared in a concentration of 10 ml/kg, and the drugs used in the combination were prepared in a concentration of 5 ml/kg each to maintain a 1 μl/g volume of administration in all groups. Ketamine (Ketamax-50) was purchased from Troikaa Pharmaceuticals Ltd., Gujarat, India, while xylazine (Xyla-Ject®) was purchased from Adwia Co. (Cairo, Egypt).

### Induction of hepatic I/R injury

Since female rats exhibit greater susceptibility to hepatic I/R injury (Gasbarrini et al. 2001) and faster post-ischemic recovery than males (de Vries et al. 2013), they were selected for this study as a more sensitive model for evaluating hepatoprotective effects. Following an overnight fasting, a ketamine/xylazine cocktail (100/10 mg/kg, i.p. (Atef et al. 2017)) was used to anesthetize all rats. Before making an incision, the abdominal area was disinfected with 70% ethanol after shaving, and then an abdominal midline laparotomy was done with the application of betadine® solution. The portal vein and hepatic artery were clamped at the median and left lateral lobes of the liver using Micro Bulldog clamps to induce 70% partial ischemia. The other 30% of the liver mass was maintained normally perfused with blood supply to prevent intestinal congestion. After 1 h of ischemia (M Hamed et al. 2018), the clamps were removed, and reperfusion was permitted for 6 h. The sham group was subjected to laparotomy only.

### Experimental design

Thirty-six rats were randomly allocated to six groups (*n* = 6/group). Group I was designated as the sham group, where rats received saline for 3 consecutive days and were exposed to laparotomy only. Group II was designated as the I/R group, where rats were administered saline for 3 consecutive days and subjected to I/R surgery. Groups III, IV, and V were designated as infliximab 1.5, infliximab 3, and amygdalin, where rats were injected with infliximab (1.5 mg/kg) (Zălar et al. 2021), infliximab (3 mg/kg) (Yucel et al. 2015; Akdogan et al. 2016; Hassan et al. 2023), or amygdalin (5 mg/kg) (Elased et al. 2020; Zhang et al. 2022), respectively. Finally, group VI received a combination of infliximab 1.5 + amygdalin. All treatments were given by intraperitoneal injection and administered for 3 consecutive days before the induction of I/R.

Low doses of both agents were deliberately selected for the combination treatment group to better reflect clinically achievable plasma concentrations and to avoid toxic responses. Using submaximal doses also facilitates the detection of potential additive or synergistic interactions between the tested compounds, providing a more accurate indication of their combined efficacy under physiologically relevant conditions.

### Biological sampling

After the 6 h of reperfusion, animals were anesthetized, and blood was collected from the abdominal aorta followed by euthanasia. The sera were separated from the centrifuged blood samples and kept at − 80 °C. Liver samples were harvested and apportioned into two sections; one section was preserved in 10% formalin for histopathological and immunohistochemical examination, and the other section was stored at − 80 °C for biochemical analysis.

### Histopathological examination and immunohistochemical analysis

Formalin preserved liver sections were paraffinized and stained by hematoxylin and eosin (H&E) stain. Sections were examined by a light microscope, and Suzuki score (0–4) was used for the quantification of sinusoidal congestion, cytoplasmic vacuolization, and parenchymal necrosis in each section (Suzuki et al. 1991). Meanwhile, liver sections were cut into adhesive slides and blocked for endogenous peroxidases after exposure to heat-induced epitope retrieval. Sections were then incubated with the primary antibodies anti-NF-κB (1:100, Santa Cruz, Biotechnology Inc., Cat# ab16502), anti-HSP90 (1:500, Proteintech, Germany, Cat#13,171–1-AP), and anti-Nrf2 (1:300, Proteintech, Germany, Cat#16,396–1-AP) for 1 h at room temperature and then washed. Universal HRP-labeled detection kit (Bio SB Inc., CA, USA) was used following the manufacturer’s guidelines. Slides that were not incubated with the primary antibody served as control. The results were computed by taking the average percentage of positive expression area from five randomly chosen non-overlapping fields in each section.

### Biochemical analysis

#### Spectrophotometric analysis

Alanine aminotransferase (ALT) and aspartate aminotransferase (AST) were measured in serum using colorimetric tests according to the provider’s instructions (Spinreact, Girona, Spain, Cat #BEIS11-E & BEIS09-E, respectively). Meanwhile, malondialdehyde (MDA) and superoxide dismutase (SOD) were measured calorimetrically in liver tissue homogenate using the kits provided by Biodiagnostic Diagnosis & Research Reagents (Giza, Egypt, Cat # MD2529, and SD2521, respectively).

#### ELISA analysis

The protein of TNF-α was determined in a 10X tissue homogenate using Rat TNF-α ELISA Kits (Cloud-Clone Corp., TX, USA, Cat#SEA133Ra) following the instructions provided by the manufacturers.

#### Western blot technique

Western blot technique was used to measure the amount of hepatic *p-MLKL*. First, the ReadyPrepTM protein extraction kit (Bio-Rad Inc., CA, USA, Cat #1,632,086) was utilized to extract total proteins from liver tissue lysates (*n* = 3) in accordance with the producer’s directions. The Bradford Protein Assay Kit (Bio Basic Inc., Ontario, Canada, Cat #SK3031) was then used to measure the protein concentration. To denature proteins, 20 μg protein from each sample was combined with two times as much Laemmli sample buffer (pH 6.8) and heated for 5 min at 95 °C. Proteins were transferred onto a nitrocellulose membrane using the Trans-Blot Turbo Transfer System (Bio-Rad Laboratories, Dubai, UAE) after being separated by molecular weight using SDS-PAGE and the TGX Stain-FreeTM FastCastTM Acrylamide Kit (Bio-Rad Laboratories, Dubai, UAE, Cat #1,610,185). TBST buffer containing 3% BSA was then used to block the membrane for 1 h at room temperature. After that, it was incubated with the *p-MLKL* primary antibody for an entire night at 4 °C. After washing with TBST, the membrane was incubated for 1 h at room temperature with an HRP-conjugated secondary antibody Goat anti-rabbit IgG-HRP (Novus Biologicals, CO, USA, Cat# NB7187). The chemiluminescent substrate (Clarity™ Western ECL; Bio-Rad Laboratories, Dubai, UAE) was applied, and signals were detected using a CCD camera-based imager. Band intensities were quantified using ChemiDoc MP imager software after normalization against β-actin as a housekeeping protein.

### Estimation of drug interaction

Drug interaction was estimated using the coefficient of drug index (CDI) according to the following equation (El-Nasr et al. 2020): CDI = AB/(A × B).

Where:

$$\mathrm{A}=\frac{\text{Mean of Inflix}. 1.5}{\text{Mean of I}/\mathrm{R}}\text{ B}=\frac{\text{Mean of Amygdalin}}{\text{Mean of I}/\mathrm{R}}\text{ AB}=\frac{\text{Mean of Inflix }1.5+\mathrm{Amygdalin}}{\text{Mean of I}/\mathrm{R}}$$

The results were interpreted as either synergistic (< 1), additive = 1, or antagonistic (>) 1 interaction.

### Assessment of correlation between variables

Data from all groups was collectively analyzed (*n* = 18 for *p-MLKL*, *n* = 36 for TNF-α, MDA, and SOD and *n* = 15 × 3 = 90 for Nrf2, NF-кB, and HSP90) using Pearson’s correlation analysis after passing the normality test using Shapiro–Wilk test (*p* > 0.05), where *r* = 1 indicates perfect positive correlation, whereas *r* = − 1 indicates perfect negative correlation. Level of significance was set at *p* < 0.05.

#### Statistical analysis

All statistical analysis and attached graphs were generated using GraphPad Prism version 8.0 (GraphPad Prism Software, CA, USA). Mean ± standard deviation (SD) was used to express values for parametric data while median (min–max) for nonparametric ones, where *n* refers to individual animals per group, each contributing one independent biological replicate to the analysis. Normally distributed parametric and non-parametric (scores) data were analyzed using one-way analysis of variance (ANOVA) followed by Tukey’spost hoc test or Kruskal–Wallis and Dunn’s multiple comparison test, respectively. Data which failed normality test (AST) were analyzed using Kruskal–Wallis and Dunn’s multiple comparison test or Mann–Whitney test for comparison between two groups. The significant level was set at *p* < 0.05.

## Results

### Amygdalin and/or infliximab improved liver function and attenuated oxidative stress

**Fig. 1:**
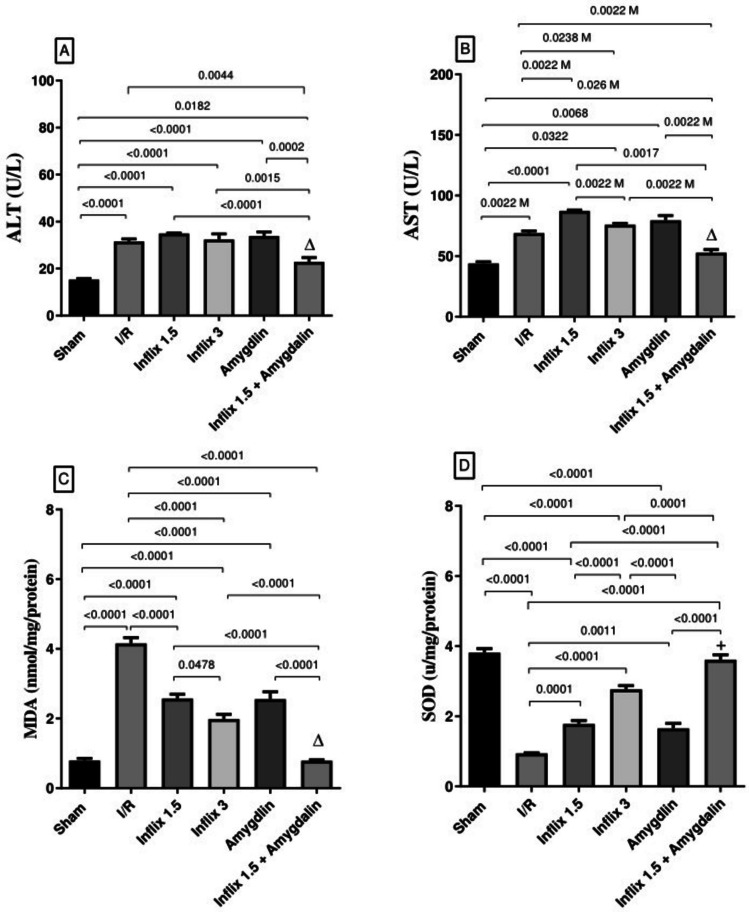
Amygdalin and/or infliximab improved liver function and attenuated oxidative stress. Rats were pretreated with either infliximab (1.5 or 3 mg/kg, i.p), amygdalin (5 mg/kg, i.p), or a combination of infliximab (1.5 mg/kg) and amygdalin for 3 days. Data is expressed as mean ± SD (*n* = 6); ALT, MDA, and SOD were analyzed using one-way ANOVA followed by Tukey’s multiple comparison test, while AST (not normally distributed) was analyzed using Kruskal–Wallis analysis of variances test followed by Dunn’s multiple comparison test or Mann–Whitney test for comparison between two groups. **M** means analyzed using Mann–Whitney test; (∆) and (+) indicate synergism and addition respectively. ALT, alanine transaminase; AST, aspartate transaminase; I/R, ischemia/reperfusion; MDA, malondialdehyde; SOD, superoxide dismutase

Figure 1 demonstrates that hepatic I/R injury resulted in a significant elevation in serum levels of (A) ALT (105.75%, *p* < *0.0001*) and (B) AST (57.06%, *p* = *0.0022 *usingMann–Whitneytest) compared to the sham-operated group. Pretreatment with the combination of infliximab (1.5 mg/kg) and amygdalin mildly attenuated this increase, reducing ALT and AST levels by 27.6%, *NS*, and 23.3%, *p* = *0.0022 *using Mann–Whitneytest, respectively. Additionally, hepatic I/R induced a pronounced increase in hepatic (C) MDA content (426.08%, *p* < *0.0001*) and a marked depletion in (D) SOD activity (75.1%, *p* < *0.0001*) relative to sham controls. Both infliximab (1.5 mg/kg) and amygdalin monotherapies produced moderate improvements, reducing MDA levels by 38.1% (*p* < *0.0001*) and 38.6% (*p* < *0.0001*) and restoring SOD levels by 53.4% (*p* < *0.0001*) and 56.6% (*p* = *0.0011*), respectively. A higher dose of infliximab (3 mg/kg) exerted greater effects causing a 52.3% (*p* < *0.0001*) reduction in MDA and a 194.8% (*p* < *0.0001*) increase in SOD, while the combined regimen achieved the most pronounced outcomes, with an 81.1% (*p* < *0.0001*) reduction in MDA and a 284.8% (*p* < *0.0001*) enhancement in SOD activity.

### Infliximab and/or amygdalin reduced the hepatic content of TNF-α, p-MLKL and enhanced the content of Nrf-2

**Fig. 2:**
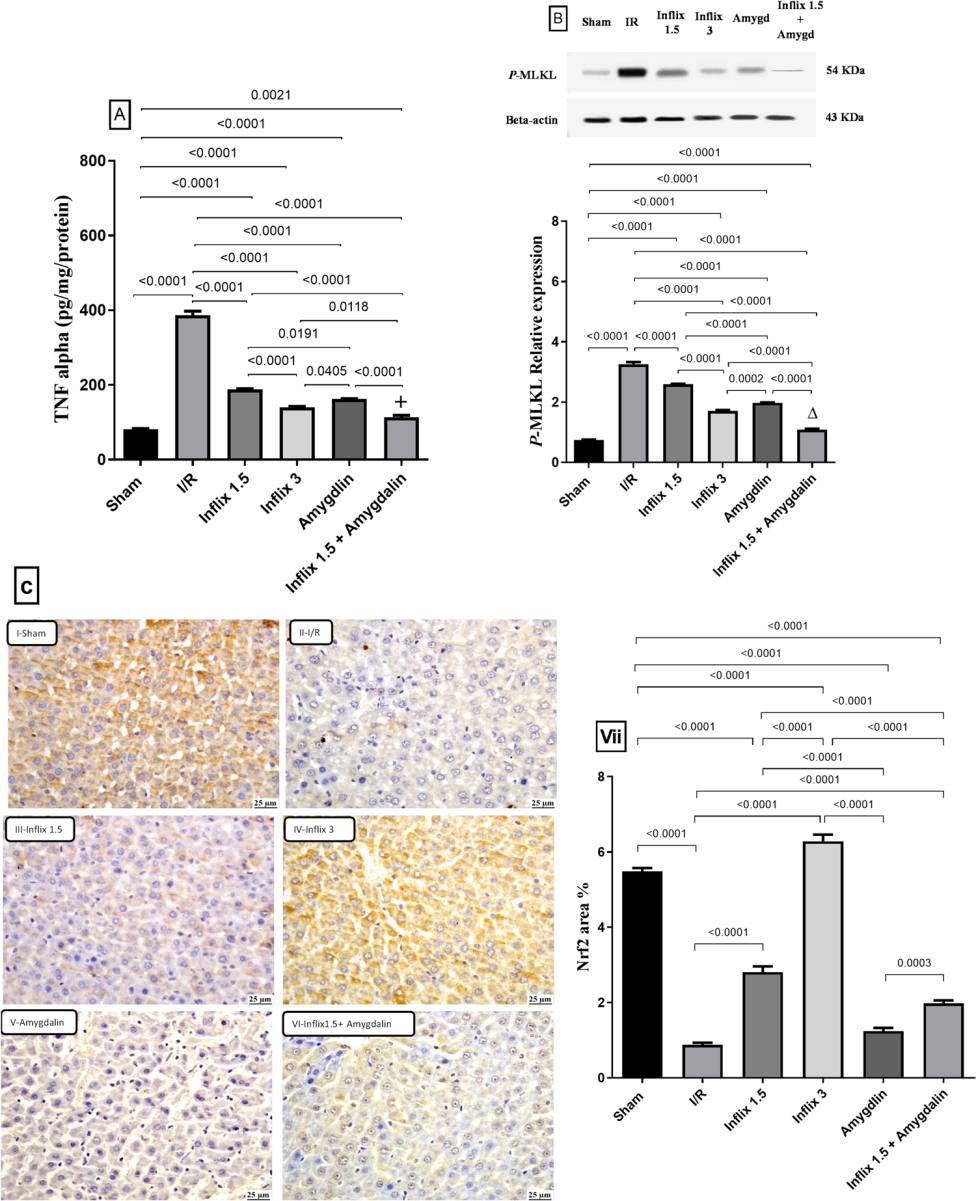
Infliximab and/or amygdalin reduced the hepatic content of TNF-α, *p-MLKL* and enhanced the content of Nrf-2. Rats were pretreated with either infliximab (1.5 or 3 mg/kg, i.p.), amygdalin (5 mg/kg, i.p.), or a combination of infliximab (1.5 mg/kg) and amygdalin for 3 days. All data for **A** TNF-α (*n* = 6), **B** *p-MLKL* (*n* = 3), and **C** Nrf2 (*n* = 3) are expressed as mean ± SD and analyzed using one-way ANOVA followed by Tukey’s multiple comparison test. (∆) and (+) indicate synergism and addition respectively. Inflix, infliximab; I/R, ischemia/reperfusion; Nrf2, nuclear factor erythroid-related factor2; *p-MLKL*, mixed lineage kinase domain-like; TNF-α, tumor necrosis factor alpha (scale bar: 25 μm)

Figure 2 illustrates that hepatic I/R injury induced a marked pro-inflammatory response, as evidenced by a 372.44% (*p* < *0.0001*) increase in hepatic TNF-α levels compared to the sham group. Pretreatment with infliximab at 1.5 mg/kg, infliximab at 3 mg/kg, and amygdalin reduced TNF-α levels by approximately 51.4%, 63.9%, and 57.9% (*p* < *0.0001*), respectively. Similarly, I/R injury led to a 334.27% (*p* < *0.0001*) increase in hepatic *p-MLKL*, a terminal mediator of necroptosis. Pretreatment with infliximab 1.5 mg/kg, infliximab 3 mg/kg, and amygdalin attenuated *p-MLKL* levels by 20.5%, 47.5%, and 39.2% (*p* < *0.0001*), respectively. Notably, the combination of infliximab 1.5 mg/kg and amygdalin resulted in a 70.7% (*p* < *0.0001*) reduction in TNF-α (*p* < *0.0001)* along with a 66.6% reduction in *p-MLKL* levels, suggesting a synergistic inhibitory effect on necroptotic signaling.

In parallel, (C) histological analysis of liver sections revealed that partial hepatic I/R insult caused an 84% (*p* < *0.0001*) depletion in Nrf2 expression relative to the sham group. Treatments with infliximab 1.5 mg/kg and infliximab 3 mg/kg increased Nrf2 levels by 221.6% and 618.9% (*p* < *0.0001*), respectively, compared to the I/R group. However, the effect of amygdalin was not significant. The combination of infliximab 1.5 mg/kg with amygdalin, on the other hand, resulted in a moderate 126.3% (*p* < *0.0001*) increase in Nrf2 levels. Infliximab 3 mg/kg showed the best effect in enhancing the antioxidant response, where it completely replenished the hepatic content of Nrf2.

### Infliximab and/or amygdalin inhibited the expression of NF-кB

**Fig. 3:**
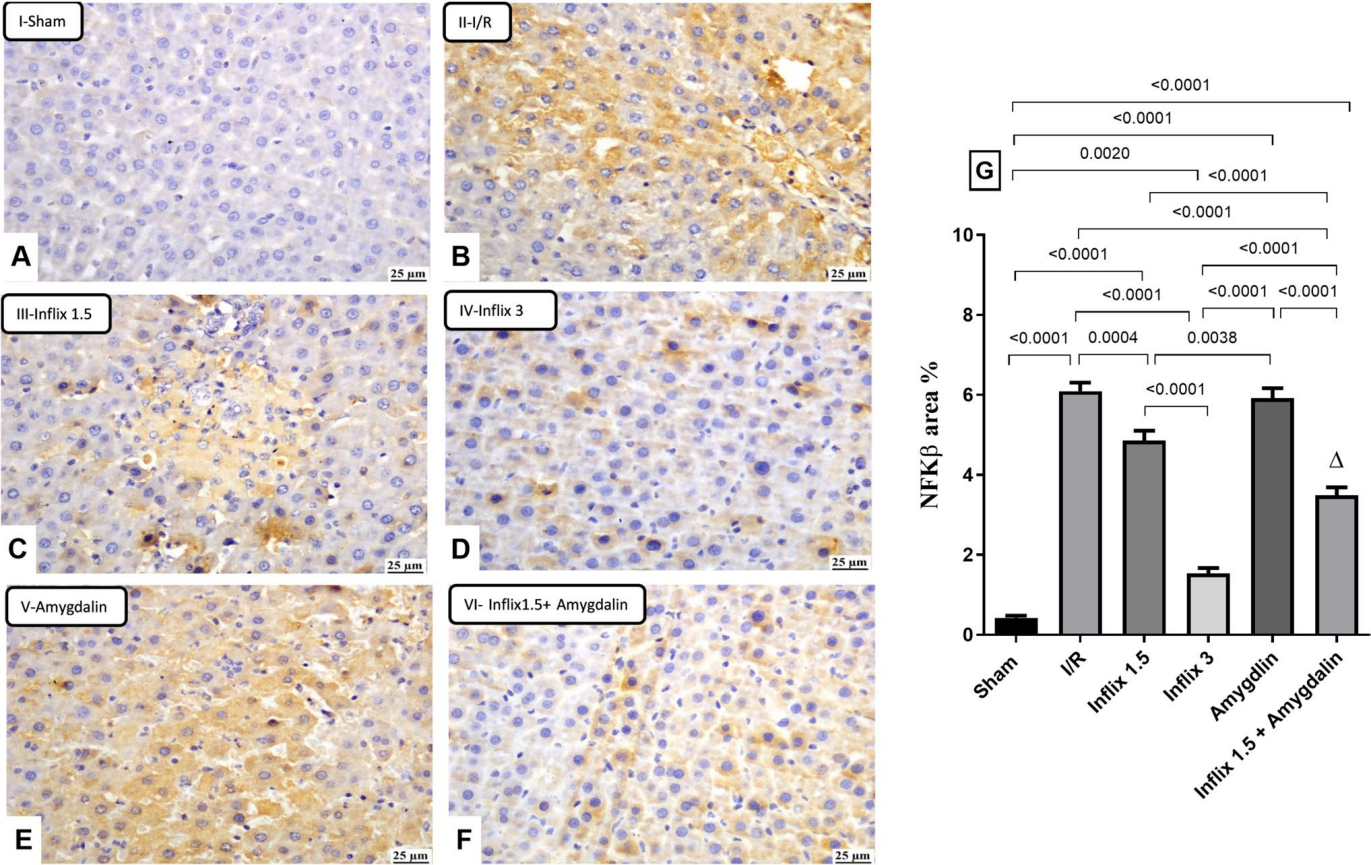
Infliximab and/or amygdalin inhibited the expression of NF-кB. Rats were pretreated with either infliximab (1.5 or 3 mg/kg, i.p), amygdalin (5 mg/kg, i.p), or a combination of infliximab (1.5 mg/kg) and amygdalin for 3 days. Data are expressed as mean ± SD (*n* = 3) and analyzed using one-way ANOVA followed by Tukey’s multiple comparison test. (∆) indicates synergism. I/R, ischemia/reperfusion; NF-кB, nuclear factor kappa B (scale bar: 25 μm)

Figure 3 presents the immunohistochemical analysis of liver sections, revealing that partial warm hepatic I/R significantly upregulated NF-κB expression by 1327.5% (*p* < *0.0001*) compared to the sham group. Pretreatment with infliximab at 1.5 mg/kg and infliximab at 3 mg/kg reduced NF-κB expression by approximately 20.3% (*p* = *0.0004*) and 74.7% (*p* < *0.0001*), respectively, while the effect of amygdalin was non-significant (2.9%). The combination of infliximab 1.5 mg/kg and amygdalin further reduced NF-κB expression by 42.8% (*p* < *0.0001*) relative to the I/R group, indicating a synergistic effect that partially mitigated the pro-inflammatory impact of the I/R insult. Among all treatment groups, infliximab 3 mg/kg demonstrated the most pronounced suppression of NF-κB expression.

### Infliximab and/or amygdalin reduced the hepatic content ofthe HSP90

**Fig. 4:**
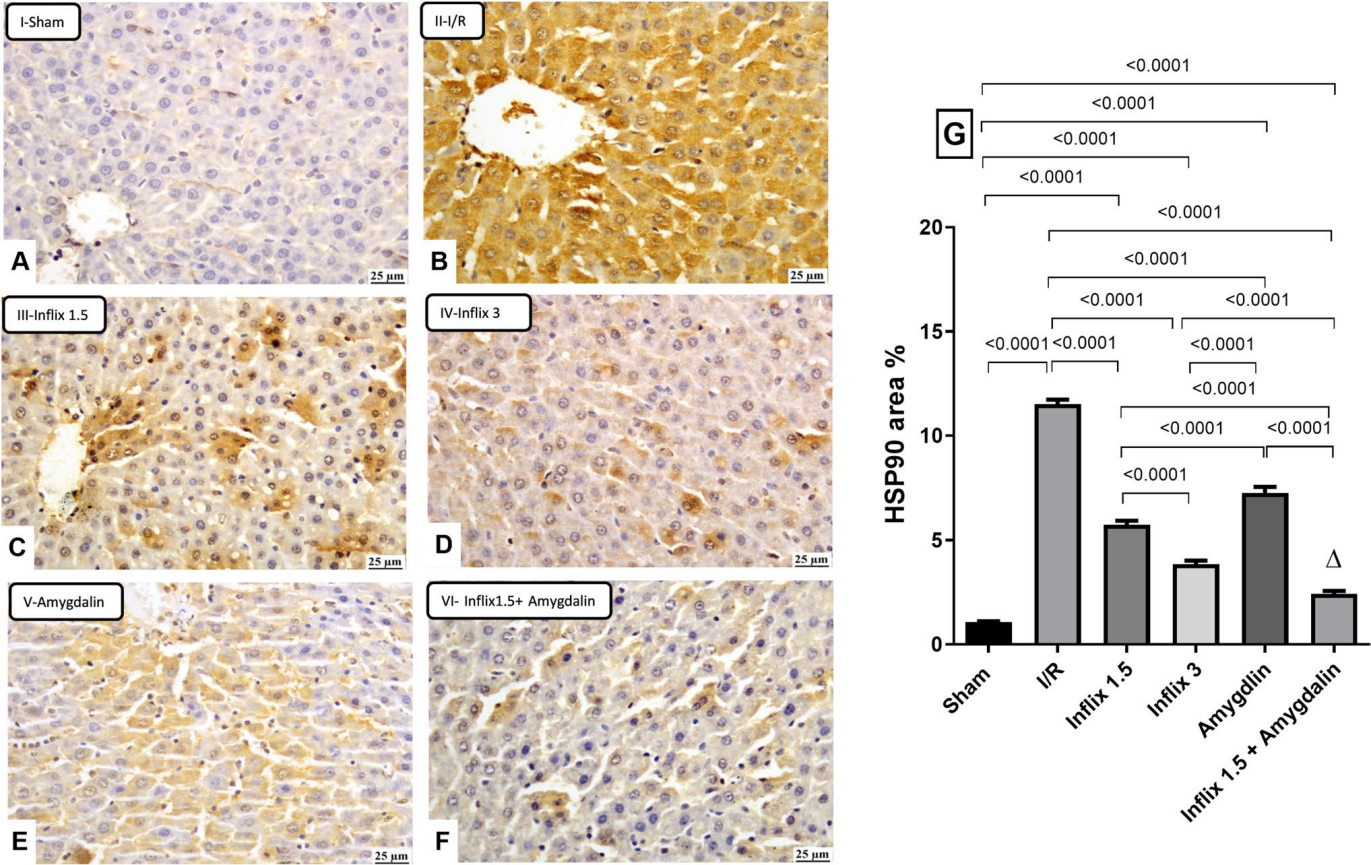
Infliximab and/or amygdalin reduced the hepatic content ofthe HSP90. Rats were pretreated with either infliximab (1.5 or 3 mg/kg, i.p), amygdalin (5 mg/kg, i.p), or a combination of infliximab (1.5 mg/kg) and amygdalin for 3 days. Data is expressed as mean ± SD (*n* = 3) and analyzed using one-way ANOVA followed by Tukey’s multiple comparison test. (∆) indicates synergism. I/R, ischemia/reperfusion; HSP90, heat shock protein 90 (scale bar: 25 μm)

As shown in Fig. 4, hepatic I/R insult triggered a pronounced elevation in HSP90 protein expression, reaching a 981.8% (*p* < *0.0001*) increase relative to the sham group. This stress-induced upregulation was notably attenuated by all treatment regimens. Infliximab at 1.5 mg/kg and 3 mg/kg reduced HSP90 levels by 50.2% and 66.6% (*p* < *0.0001*), respectively, while amygdalin alone achieved a more modest reduction of 36.8% (*p* < *0.0001*). Interestingly, the combination of infliximab 1.5 mg/kg with amygdalin produced a more pronounced suppression (79.13%, *p* < *0.0001*), highlighting a potential synergistic interaction in downregulating HSP90 in the context of I/R injury.

### Amygdalin and/or infliximab improved the histopathological picture

**Fig. 5:**
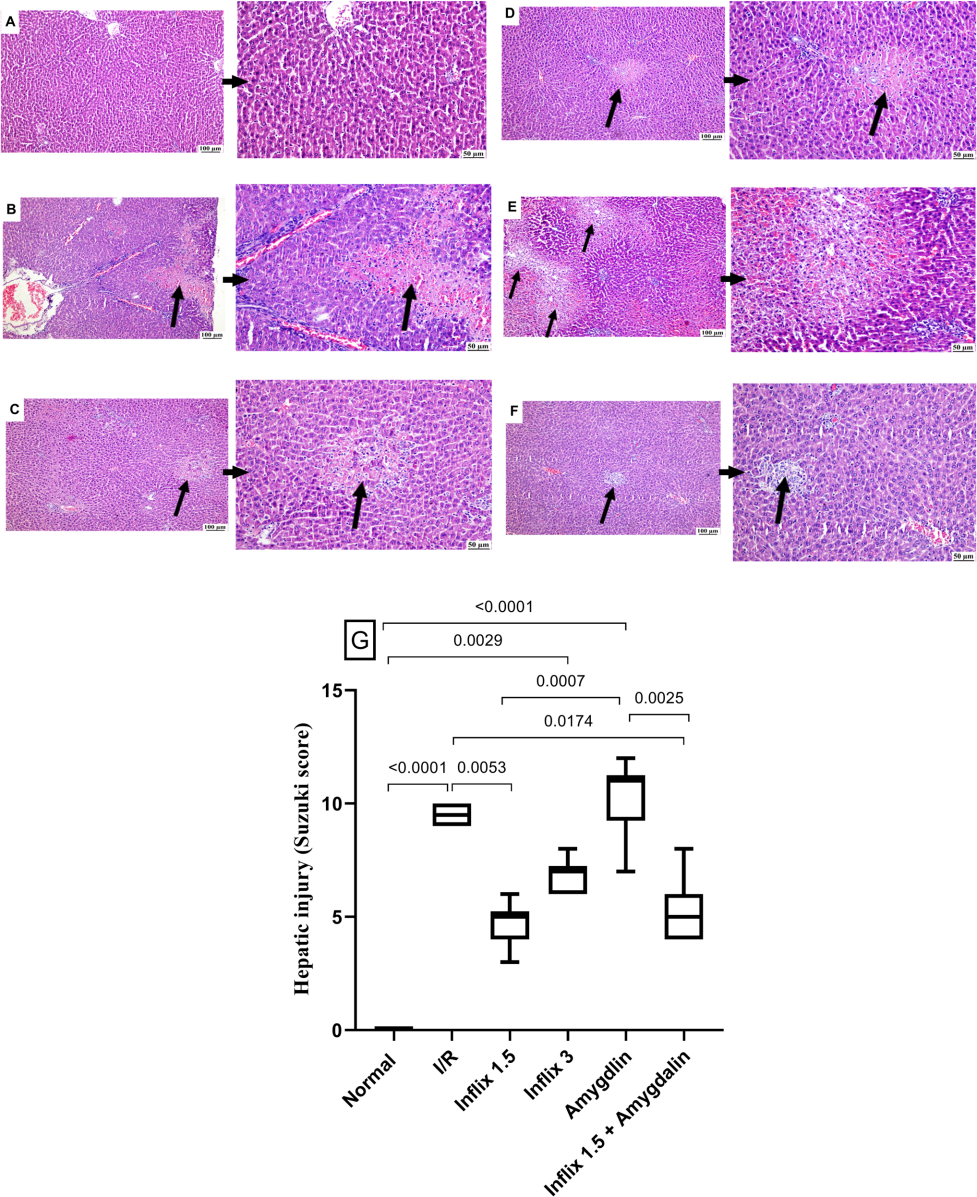
Amygdalin and/or infliximab improved the histopathological picture. Rats were pretreated with either infliximab (1.5 or 3 mg/kg, i.p), amygdalin (5 mg/kg, i.p.), or a combination of infliximab (1.5 mg/kg) and amygdalin for 3 days. Scores are expressed as median (min–max) (*n* = 3) and analyzed by using the Kruskal–Wallis analysis of variances test followed by Dunn’s multiple comparison test. I/R, ischemia/reperfusion (scale bar: 100 μm and 50 μm)

Histological sections of liver tissue from the various experimental groups showed the normal liver architecture in (Fig. 5A) the sham-operated group, with no discernible histopathological alterations. In contrast, the I/R group (Fig. 5B) exhibited extensive hepatic damage characterized by widespread hepatocellular necrosis, pronounced neutrophilic infiltration, severe vascular congestion, and focal hemorrhagic areas, hallmarks of acute hepatic injury. Treatment with infliximab at 1.5 mg/kg (Fig. 5C) markedly ameliorated these pathological changes, as evidenced by limited focal necrosis and a predominance of mononuclear inflammatory cells rather than neutrophils. A similar histological pattern was observed in the infliximab 3 mg/kg group (Fig. 5D), where scattered necrotic foci and portal mononuclear cell infiltration were evident. On the other hand, the amygdalin-treated group (Fig. 5E) showed less protection, with sections displaying large necrotic zones and marked sinusoidal dilatation. Remarkably, the combination therapy group (Fig. 5F) demonstrated near-complete preservation of hepatic architecture in several specimens, with only minimal mononuclear cell aggregation in others, indicating substantial histological recovery. These microscopic observations were corroborated by Suzuki histological scoring (Fig. 5G), which quantitatively supported the protective effects of the treatments, particularly the combination regimen.

### Correlation between variables

**Fig. 6:**
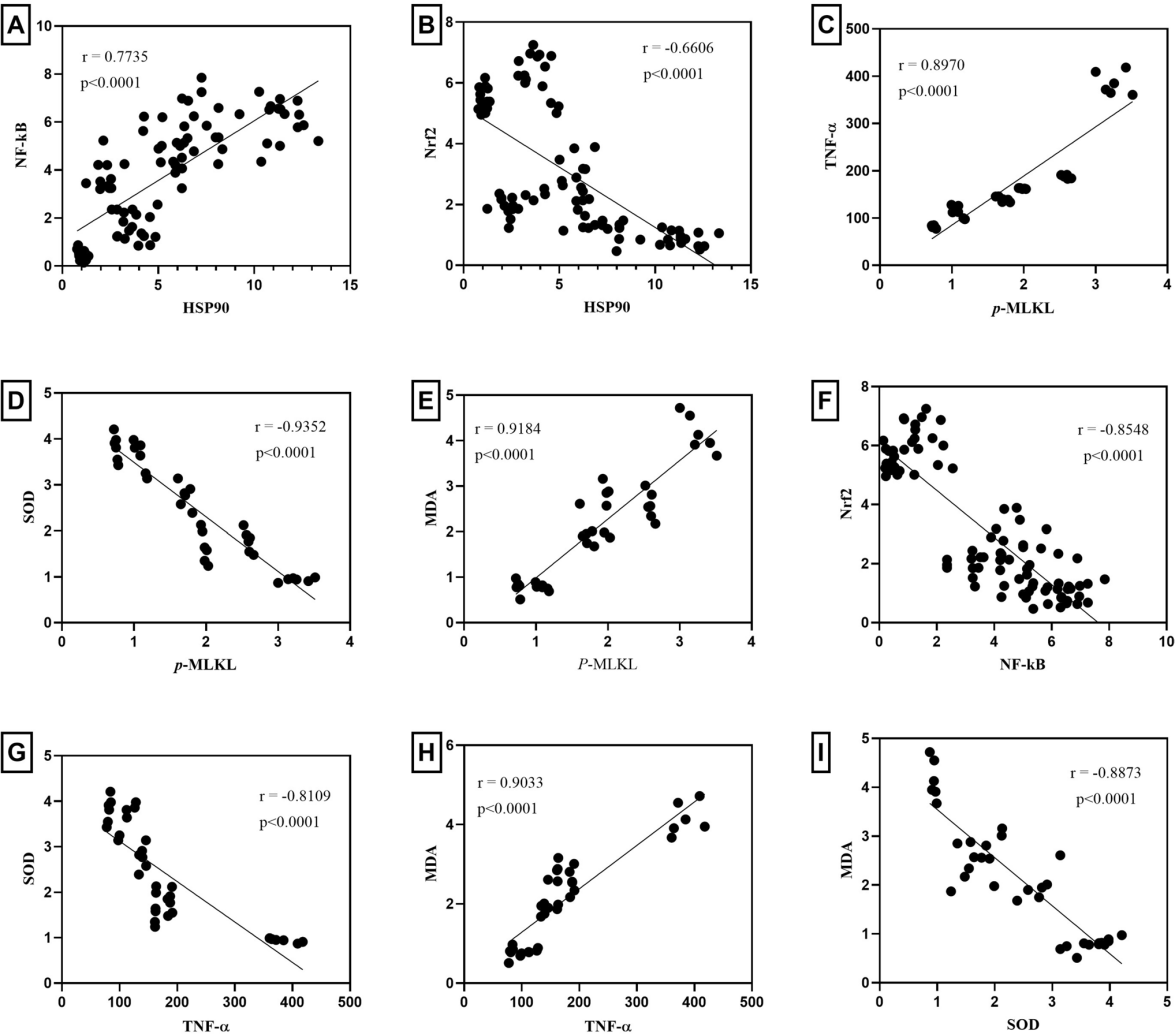
Correlation between the different variables. Data from all groups was collectively analyzed using Pearson’s correlation analysis. A positive correlation was observed between **A** HSP90 and NF-κB, **C** *p-MLKL* and TNF-α, **E** *p-MLKL* and MDA in addition to **H** TNF-α and MDA. Meanwhile, a negative correlation was detected between **B** HSP90 and Nrf2, **D** *p-MLKL* and SOD, **F** NF-κB and Nrf2, **G** TNF-α and SOD, as well as **I** SOD and MDA. HSP90, heat shock protein 90; MDA, malondialdehyde; NF-κB, nuclear factor kappa B; Nrf2, nuclear factor erythroid-related factor2; *p-MLKL*, mixed lineage kinase domain-like; SOD, superoxide dismutase; TNF-α, tumor necrosis factor alpha

Figure 6 summarizes the correlation between the different markers in the current study as assessed by Pearson’s correlation analysis. A positive correlation was observed between HSP90 and NF-κB (*r* = 0.7735, *p* < 0.0001), p*-MLKL* and TNF-α (*r* = 0.8970, *p* < 0.0001), *p-MLKL* and MDA (*r* = 0.9184, *p* < 0.0001), and TNF-α and MDA (*r* = 0.9033, *p* < 0.0001). Meanwhile, a negative correlation was detected between HSP90 and Nrf2 (*r* = 0.6606, *p* < 0.0001), *p-MLKL* and SOD (*r* = − 0.9352, *p* < 0.0001), NF-кB and Nrf2 (*r* = − 0.8548, *p* < 0.0001), TNF-α and SOD (*r* = − 0.8109, *p* < 0.0001), as well as SOD and MDA (*r* = − 0.8873, *p* < 0.0001).

## Discussion

**Fig. 7:**
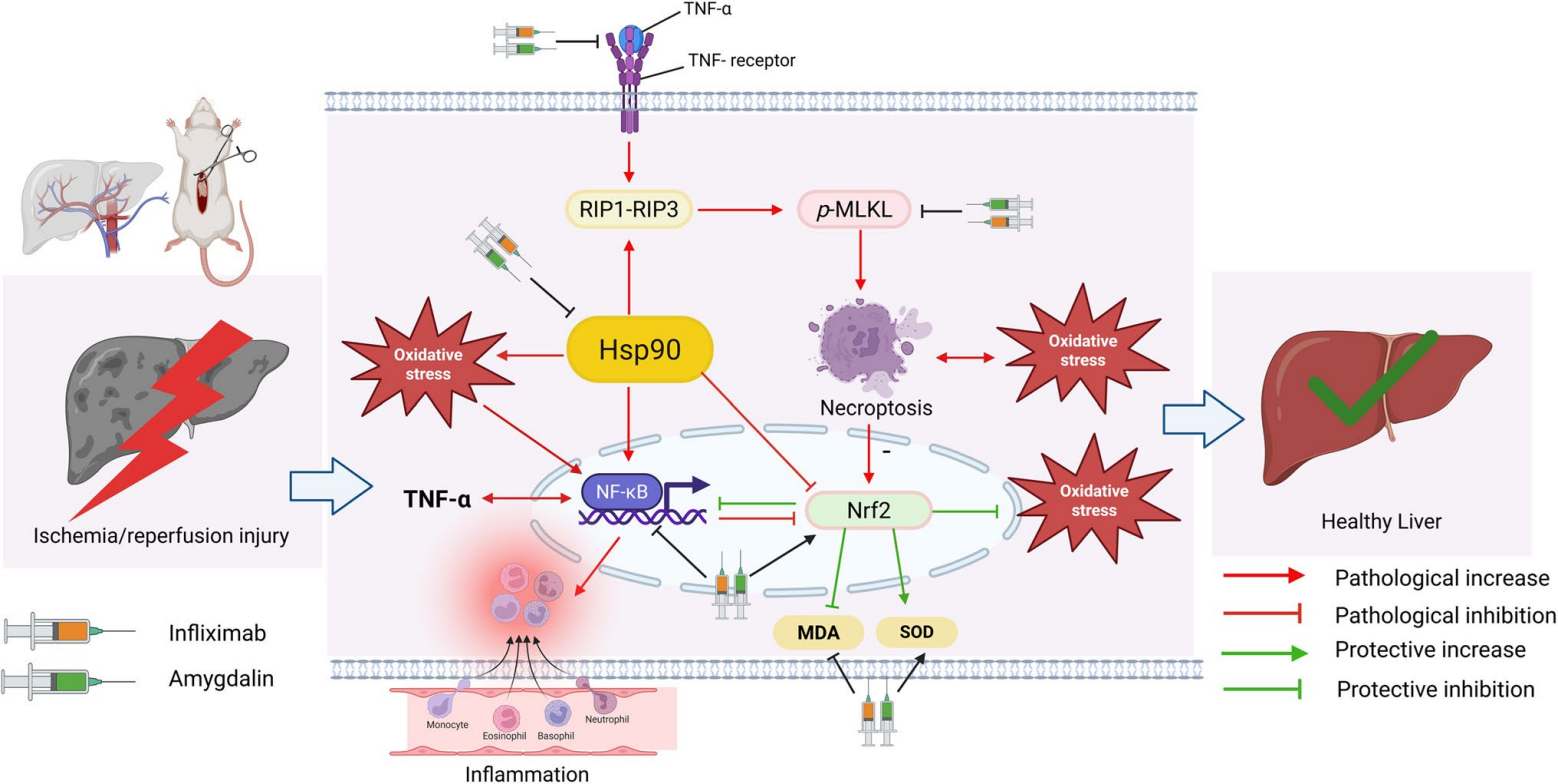
Proposed mechanism underlying the protective effects of the amygdalin and infliximab combination against hepatic ischemia/reperfusion (I/R) injury. Hepatic I/R triggers excessive tumor necrosis factor (TNF)-α release, which activates TNF receptors and subsequently induces mixed lineage kinase domain-like protein (MLKL) phosphorylation and receptor-interacting protein kinase 1 (RIP1)/receptor-interacting protein kinase 3 (RIP3) signaling, thereby promoting necroptosis and amplifying oxidative stress. Concurrently, TNF-α activates the nuclear factor kappa B (NF-κB) pathway, driving inflammation while suppressing the liver’s antioxidant defenses through inhibition of nuclear factor erythroid 2–related factor 2 (Nrf2). These pathological processes are further exacerbated by increased heat shock protein 90 (Hsp90) activity, contributing to additional oxidative damage. The amygdalin and infliximab combination mitigates I/R-induced injury by inhibiting TNF-α/NF-κB signaling, suppressing Hsp90 and RIP1/RIP3/MLKL-mediated necroptosis, and restoring antioxidant capacity via Nrf2 activation (↓ malondialdehyde (MDA), ↑ superoxide dismutase (SOD)). Collectively, these actions reduce inflammation, oxidative stress, and programmed necrosis, ultimately preserving hepatic integrity

In the current study, pre-treatment with infliximab (1.5 and 3 mg/kg), amygdalin (5 mg/kg), and their combination (infliximab 1.5 + amygdalin) ameliorated the pathological perturbations caused by the hepatic I/R injury, where all treatment regimens reduced the hepatic content of TNF-α, the necroptosis marker *p-MLKL*, NF-κB (except amygdalin), and HSP90 along with a decrease in oxidative stress (↓ MDA, ↑ SOD) and increase in Nrf2 (except amygdalin) and finally improvement in the histopathological picture. The effect of the combination regimen was synergistic on some markers (ALT, AST, *p-MLKL*, NF-κB, HSP90, and MDA) and additive in others (SOD and TNF-α) as compared to infliximab or amygdalin monotreatment. The mechanistic pathway is illustrated in Fig. 7.

Amygdalin exhibits anti-inflammatory and anti-tumor activities by modulating apoptosis, cell proliferation, metastasis, and inflammation (He et al. 2020; Figurová et al. 2021). In liver injury, it shows hepatoprotective effects by reducing hepatic damage and inflammatory cell infiltration (Elsaed 2019; Wang et al. 2021), though its role in hepatic I/R injury was not previously investigated. Meanwhile, infliximab, a TNF-α inhibitor, improves liver function, reduces inflammation, and mitigates oxidative stress in hepatic I/R (Yucel et al. 2015). Thus, combining amygdalin with infliximab may provide additive/synergistic benefits by targeting complementary pathological pathways.

Necroptosis is a key pathological pathway in I/R injury, activated when apoptosis is blocked, such as during viral infection or by cellular inhibitors of apoptosis protein (cIAP1/2) (Field and Gordon 2022). This process involves RIP1, RIP3, and MLKL, where TNF-α binding to its receptor initiates RIPK1/RIPK3 necrosome formation and subsequent MLKL phosphorylation. Activated *p-MLKL* oligomerizes at the plasma membrane, forming pores that lead to lytic cell death and the release of DAMPs and cytokines, thereby amplifying inflammation (Ashkenazi and Salvesen 2014; Vanden Berghe et al. 2016; Liu et al. 2017; Kim et al. 2019). In this study, infliximab, amygdalin, and their combination reduced hepatic TNF-α and *p-MLKL* expression. The inhibitory effects of amygdalin on TNF-α and *p-MLKL* were previously shown in carrageenan-induced arthritis (Hwang et al. 2008) and acetaminophen-induced liver failure (Zhang et al. 2022), while infliximab demonstrated similar activity in ischemic stroke (Chen et al. 2019).

Moreover, hypoxia followed by reoxygenation in I/R accelerates reactive oxygen species (ROS) formation and oxidative stress, which trigger necroptosis (Berghe et al. 2014) and activate proinflammatory cytokines and chemokines (El Sayed et al. 2021), leading to acute inflammation and hepatocellular injury (George et al. 2024). On the other hand, necroptosis further amplifies oxidative stress by suppressing Nrf2 (Zhang et al. 2021), via RIP1 signaling (Li et al. 2021a), and through RIP3-mediated phosphorylation of pyruvate dehydrogenase complex, which enhances mitochondrial ROS production (Yang et al. 2018). Thus, necroptosis functions as an upstream driver of oxidative stress in hepatic I/R (Jia et al. 2018; Song and Li 2019). Under basal conditions, Nrf2 is bound to its cytoplasmic inhibitor protein Kelch-like ECH-associated protein 1 (Keap1), rendering it inactive, but during oxidative stress it dissociates and translocates to the nucleus, where it dimerizes with a small musculoaponeurotic fibrosarcoma oncogene homolog (Maf) protein to activate antioxidant response elements, thereby inducing genes such as glutathione (Stefanson and Bakovic 2014), heme oxygenase-1 (HO-1) (Jian et al. 2011), and SOD (Tu et al. 2019; Yi et al. 2024). Necroptosis suppresses Nrf2, as shown by studies where necroptosis inhibitors necrostatin-1 and GlaxoSmithKline-872 reduced ROS levels (Yang et al. 2019). Consistently, the present study demonstrated that I/R triggered necroptosis as evidenced by increased *p-MLKL* expression while decreasing hepatic Nrf2 and worsening oxidative stress (↑MDA, ↓SOD). In contrast, infliximab (both doses) and its combination with amygdalin reduced *p-MLKL*, restored Nrf2, and improved oxidative balance, with the combination fully normalizing MDA and SOD.

Additionally, inflammatory and oxidative responses are co-regulated by Nrf2 and NF-κB, where Nrf2 deficiency enhances NF-κB activity and inflammatory mediator production, while NF-κB negatively regulates Nrf2 and its downstream targets (Gao et al. 2022). Their crosstalk may involve competition for binding to cAMP response element-binding protein (CREB)-binding proteins in the nucleus, and NF-κB-recruited histone deacetylases that inactivate Nrf2. Furthermore, NF-κB inhibitors can indirectly activate Nrf2; similarly, Nrf2 inhibitors cause the indirect activation of NF-κB. Even more, downstream proteins of Nrf2 such as HO-1 can inhibit NF-κB transcription (Gao et al. 2022), pointing to their reciprocal regulation. In the current study, amygdalin had no effect on NF-κB or Nrf2, whereas infliximab dose-dependently modulated both, with 3 mg showing superior effects, and the combination regimen producing mild improvement. This reciprocal relationship between TNF-α and NF-κB is consistent with established evidence that TNF-α activates NF-κB via IκB degradation and nuclear translocation, while NF-κB stimulates the transcription of several pro-inflammatory genes, including TNF-α itself (Hayden and Ghosh 2014; Kagoya et al. 2014), creating a positive feedback loop relevant in I/R injury (Mahmoud et al. 2012).

To further explore modulation of necroptosis, we examined treatment effects on HSP90, a stress-induced molecular chaperone that assists protein folding and repair (Szyller and Bil-Lula 2021). HSP90 regulates RIP3 and MLKL stability and function (Li et al. 2015); the deficiency of HSP90 terminates its stabilizing effect and results in the recognition of the client proteins by the ubiquitin–proteasome pathway and their consequent degradation (Yang and He 2016). Additionally, HSP90 disruption prevents TNF-α-induced necrosis, RIP1-dependent NF-κB activation, necrosome formation, and RIP3 phosphorylation, shifting cell death toward apoptosis (Lewis et al. 2000; Berghe et al. 2003; Li et al. 2015). Conversely, increased HSP90 activity enhances MLKL oligomerization and membrane translocation (Zhao et al. 2016), underscoring its regulatory role in necroptosis (Yang and He 2016). Additionally, several studies have reported direct or indirect interactions between HSP90 and Nrf2, mainly through the regulation of Keap1 (Bonura et al. 2022; Ngo et al. 2022; Giacomarra et al. 2024). HSP90 stabilization of Keap1 suppresses Nrf2 activation, whereas HSP90 inhibition promotes Nrf2-dependent cytoprotective mechanisms (Lazaro et al. 2017). This provides an additional mechanistic basis for the observed modulation of Nrf2 and oxidative stress markers in the current study.

Beyond necroptosis and the regulation of anti-oxidant defense response, HSP90 inhibitors exert anti-inflammatory effects in arthritis (Conte et al. 2015), colitis (Collins et al. 2013), lung inflammation (Lilja et al. 2015), and other models (Costa et al. 2020), partly because several HSP90 clients such as NF-κB are important signaling factors in inflammation; hence, the loss of HSP90 activity inhibits the activation of NF-κB and inflammatory mediators to arrest inflammation (Costa et al. 2020). Furthermore, the inhibition of HSP90 inhibited TNF-α, an upstream trigger of necroptosis, in a murine intestinal inflammation model (Collins et al. 2014), providing another possible link between HSP90 activity and the activation of necroptosis. In the present study, hepatic I/R increased HSP90 alongside *p-MLKL* and NF-κB, while all treatments reduced HSP90 in ascending order: amygdalin < infliximab 1.5 < infliximab 3 < combination, the latter producing the most pronounced effect.

The modulation of HSP90 has also been implicated in clinical hepatology, underscoring its translational relevance. HSP90β is overexpressed in patients with non-alcoholic fatty liver disease and in obese mice, where its expression correlates with elevated serum lipid levels (Zheng et al. 2019). In hepatocellular carcinoma, HSP90α promotes lipogenesis by stabilizing fatty acid synthase and enhancing its transcription, thereby contributing to tumor progression and poor prognosis (Deng et al. 2025). Moreover, HSP90 inhibition, accompanied by heat shock factor 1 (HSF1) and heat shock protein family A member 1 A (HSPA1A) activation, has been shown to reduce interleukin (IL)−1β and IL-18 production by suppressing NOD-like receptor family pyrin domain–containing protein 3 (NLRP3) inflammasome, caspase-1 (CASP-1), and gasdermin D (GSDMD) activity in alcoholic liver disease (Choudhury et al. 2020). Notably, this is the first report of amygdalin and infliximab targeting HSP90. Histologically, infliximab 1.5 improved some pathological features, infliximab 3, and amygdalin induced milder changes, and the combination showed effects comparable to infliximab 1.5 mg.

Correlation analysis revealed a strong interplay among inflammation, oxidative stress, and necroptosis in hepatic I/R injury. Pro-inflammatory and pro-death markers, including HSP90/NF-κB, TNF-α/MDA, *p-MLKL*/TNF-α, and *p-MLKL*/MDA, were positively correlated, whereas negative correlations were observed with antioxidant defenses such as Nrf2 and SOD (e.g., HSP90/Nrf2, *p-MLKL*/SOD, NF-κB/Nrf2, TNF-α/SOD, SOD/MDA). Notably, HSP90 appeared to occupy a central position, showing a positive correlation with the pro-inflammatory transcription factor NF-κB and a negative correlation with the antioxidant transcription factor Nrf2. These findings highlight HSP90 as a potential mediator bridging inflammation and oxidative stress through NF-κB and Nrf2 signaling pathways.

## Conclusion, limitations, and future considerations

Amygdalin in the current study protected against hepatic I/R injury by modulating oxidative stress, inflammation, and necroptosis through mechanisms involving HSP90 and Nrf2/NF-κB signaling. Although at the used dose amygdalin had no direct effect on Nrf2 and NF-κB, its effects on these pathways appeared when combined with low dose infliximab, and its effect on the other pathways were further potentiated in combination with low-dose infliximab, suggesting a synergistic therapeutic strategy. These findings highlight amygdalin, particularly when combined with infliximab, as a promising candidate for translational applications in the management of ischemia-induced liver injury. However, the study has some limitations, including the exclusive use of female rats and the relatively short reperfusion time, which focuses on the early molecular and histopathological events of reperfusion injury and does not fully represent the chronic phases of hepatic I/R injury. Future studies should address these limitations and explore the precise molecular interactions between HSP90, NF-κB, and Nrf2 signaling pathways to better understand how HSP90 bridges inflammation and oxidative stress, potentially by using selective HSP90 inhibitors. Additionally, the potential toxic effects of amygdalin warrant thorough safety evaluation before clinical translation. Further studies are also required to optimize the doses of infliximab and amygdalin to maximize therapeutic efficacy while ensuring safety.

## Supplementary Information

Below is the link to the electronic supplementary material.

ESM1 (PDF 104 KB)

## Data Availability

All data will be available upon request.
