# Supplementary material for: Amygdalin synergizes with the TNF-α monoclonal antibody infliximab to modulate HSP90 and related necro-inflammatory/oxidative stress pathways in a rat model of hepatic I/R
Source: Naunyn Schmiedebergs Arch Pharmacol. 2025 Dec 17;399(5):7643–57. doi: 10.1007/s00210-025-04866-6 (PMC13053369; doi:10.1007/s00210-025-04866-6)
Supplement: Supplementary file 1 — (PDF 104 KB) [file 210_2025_4866_MOESM1_ESM.pdf]

| | |
|---|---|
| **Article Title** | Amygdalin synergizes with the TNF-α monoclonal antibody infliximab to modulate HSP90 and related necro-inflammatory/oxidative stress pathways in a rat model of hepatic I/R |
| **Journal name** | Naunyn-Schmiedeberg's Archives of Pharmacology |
| **Author names** | Reem A. Mohamed[a*], Mai El-Sayed Ghoneim[b], Rasha A. Tawfiq[c,d], Nermein F. El Sayed[e] |
| **Affiliation of corresponding author** | Department of Pharmacology, Faculty of Pharmacy, October University for Modern Sciences and Arts (MSA), Giza, Egypt |
| **e-mail address of corresponding author** | Ralia@msa.edu.eg, Ralia2025n@gmail.com |

| Marker | Group | | | | | | CDI | Meaning |
|---|---|---|---|---|---|---|---|---|
| **ALT** | Sham | IR | Inflix 1.5 | Inflix 3 | Amygdlin | Inflix 1.5 + Amygdalin | 0.608475 | Synergistic |
| Mean | 15.31 | 31.5 | 34.89 | 32.34 | 33.83 | 22.8 | | |
| | | | | | | | | |
| **AST** | Sham | IR | Inflix 1.5 | Inflix 3 | Amygdlin | Inflix 1.5 + Amygdalin | 0.525266 | Synergistic |
| Mean | 43.9 | 68.95 | 87.28 | 75.9 | 79.5 | 52.86 | | |
| | | | | | | | | |
| **Nrf2** | Sham | IR | Inflix 1.5 | Inflix 3 | Amygdlin | Inflix 1.5 + Amygdalin | 0.494773 | Synergistic |
| Mean | 5.481 | 0.874 | 2.811 | 6.283 | 1.243 | 1.978 | | |
| | | | | | | | | |
| **pMLKL** | Sham | IR | Inflix 1.5 | Inflix 3 | Amygdlin | Inflix 1.5 + Amygdalin | 0.690371 | Synergistic |
| Mean | 0.75 | 3.257 | 2.59 | 1.71 | 1.98 | 1.087 | | |
| | | | | | | | | |
| **Hsp90** | Sham | IR | Inflix 1.5 | Inflix 3 | Amygdlin | Inflix 1.5 + Amygdalin | 0.667914 | Synergistic |
| Mean | 1.063 | 11.5 | 5.729 | 3.841 | 7.273 | 2.42 | | |
| | | | | | | | | |
| **NFKB** | Sham | IR | Inflix 1.5 | Inflix 3 | Amygdlin | Inflix 1.5 + Amygdalin | 0.738534 | Synergistic |
| Mean | 0.4267 | 6.091 | 4.857 | 1.54 | 5.916 | 3.484 | | |
| | | | | | | | | |
| **MDA** | Sham | IR | Inflix 1.5 | Inflix 3 | Amygdlin | Inflix 1.5 + Amygdalin | 0.497999 | Synergistic |
| Mean | 0.7898 | 4.155 | 2.572 | 1.983 | 2.552 | 0.7867 | | |
| | | | | | | | | |
| **SOD** | Sham | IR | Inflix 1.5 | Inflix 3 | Amygdlin | Inflix 1.5 + Amygdalin | 1.163779 | Additive |
| Mean | 3.816 | 0.9489 | 1.78 | 2.768 | 1.655 | 3.613 | | |
| | | | | | | | | |
| **TNF-α** | Sham | IR | Inflix 1.5 | Inflix 3 | Amygdlin | Inflix 1.5 + Amygdalin | 1.433152 | Additive |
| Mean | 81.83 | 386.6 | 187.8 | 139.7 | 162.6 | 113.2 | | |
